# Comprehensive assessment of food and nutrient intake of children and adolescents in Germany: EsKiMo II – the eating study as a KiGGS module

**DOI:** 10.1186/s40795-017-0196-5

**Published:** 2017-09-12

**Authors:** C. Lage Barbosa, A.-K. Brettschneider, M. Haftenberger, F. Lehmann, M. Frank, K. Heide, E. Patelakis, H. Perlitz, L. Krause, R. Houben, H.G. Butschalowsky, A. Richter, P. Kamtsiuris, G.B.M. Mensink

**Affiliations:** Department of Epidemiology and Health Monitoring, Robert Koch Institute, General-Pape-Str. 62-66, 12101 Berlin, Germany

**Keywords:** Nutrition, Food and nutrient intake, Dietary behavior, Children and adolescents, EsKiMo, Germany

## Abstract

**Background:**

As part of the second wave of the German Health Interview and Examination Survey for Children and Adolescents (KiGGS Wave 2), food and nutrient intake of children and adolescents aged 6–17 years living in Germany is assessed in EsKiMo II – the Eating Study as a KiGGS Module.

**Methods:**

EsKiMo II is a cross-sectional study, conducted from June 2015 until September 2017. The study population comprises 6 to 17-year-old study participants from the cross-sectional sample of KiGGS Wave 2 in 167 KiGGS sample points, which are revisited by trained nutritionists. Dietary intake is assessed by weighted food records during three consecutive days plus one randomly selected day within the following 3 months for children aged 6–11 years. Dietary intake for adolescents aged 12–17 years is assessed by computer-assisted dietary history interviews, reflecting the past four weeks, using the software DISHES. Further information, for example, about specific diets and dietary supplement intake, is reported during a standardised computer assisted interview for all participants. Food items are coded by the German Food Code and Nutrient Database (BLS 3.02).

**Discussion:**

EsKiMo II provides actual data on the dietary behaviour of children and adolescents living in Germany and their determinants. Results of EsKiMo II will be relevant for decision-making, measures, and evaluations within nutrition, consumer and health policy.

## Background

The last decades have been characterized by rapid economic, social, technological, as well as demographic changes, followed by changes in dietary behavior in several countries [1, 2]. Changes in dietary behavior also seem to be driven by food trends (such as “superfoods”) [3], health campaigns, establishment of fortification of foods (e.g. vitamin D, folic acid), and technological developments [4]. In Germany, like in several other western countries, the number of food products available in the market (e.g. convenience foods, energy-dense fast foods, fortified foods, children-specific products, and vegetarian and vegan products) has increased considerably [3, 5, 6]. In addition, there have been changes in living conditions (e.g. changing of family structures, work-life balance, expansion of all-day schools) [7], dietary guidelines [8], the development and implementation of national intervention programs [9], as well as legislation and subsidies on food pricing [10–12], which might have a strong impact on behavioral patterns, as well as on dietary patterns, and, therefore, on the nutritional situation [13].

Adequate nutrition is essential for development, growth, and health of children and adolescents [14]. The regular monitoring of nutrition and dietary behavior is, consequently, especially relevant for those age groups. Children and adolescents need more nutrients than adults in relation to their body weight [15], requiring, therefore, a diet providing a higher nutrient density. Due to their lower body weight and their not yet fully developed immune system, children are also a particularly sensitive and vulnerable group for food contamination, as well as food pollution by undesirable substances, such as pesticides and acrylamide [16]. Furthermore, the energy content of the diet has a relevant impact on the development of overweight and obesity [1, 2]. From a public health point of view, this aspect is especially relevant, since children with overweight or obesity tend to persist overweight or obese during adult life [11, 17], and will consequently be at higher risk for obesity associated morbidity and mortality [11, 18–20].

Regular monitoring of nutrition status and dietary habits is important to recognize dietary deficits and possible health consequences. A regular, representative monitoring of food consumption and nutrient intake of the German population is, therefore, a relevant source for decisions making, actions, and evaluations in the field of nutrition, consumer, and health policy at both national and European level. The European Food Safety Agency (EFSA) recommends that detailed national nutrition surveys should be carried out every 8–10 years [21].

The last representative study about children’s and adolescents’ dietary behavior in Germany was conducted in 2006, as a module of the German Health Interview and Examination Survey for Children and Adolescents (KiGGS): the Eating Study as a KiGGS module (EsKiMo I) [2, 22]. It was an important source of information for both nutrition policies and consumer health protection, but since the study was conducted about 10 years ago there is an urgent need for an update. The German Federal Ministry of Food and Agriculture (BMEL) and other institutions, such as the EFSA, have a major interest in actual data of food consumption and dietary behavior, since they serve to improve consumer’s health protection of the German population. In addition, data on the nutritional situation of children and adolescents are urgently needed for current food and nutrition related policy issues.

EsKiMo II is conducted as part of KiGGS Wave 2 (2014–2017). The aim of EsKiMo II is an actual assessment of the dietary behaviour of children and adolescents aged 6–17 years living in Germany and to identify changes in food consumption in the last decade.

Design and methods of EsKiMo II are described in this paper.

## Methods/design

### Study design and study population

**Fig. 1:**
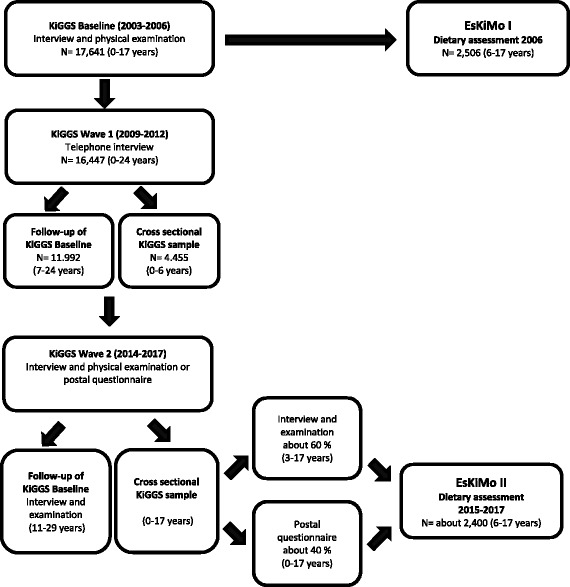
Flowchart of the study population of KiGGS and EsKiMo

The dietary surveys, EsKiMo I and II, are cross-sectional surveys, performed by the Robert Koch Institute (RKI) as part of KiGGS (Fig. 1) and funded by the BMEL. KiGGS is a nationwide representative survey on the health of children and adolescents and is part of the health monitoring system in Germany and includes three waves by now. The KiGGS baseline survey, conducted between 2003 and 2006, examined children and adolescents aged 0 to 17 years. Study design and details of the population sampling of the KiGGS baseline survey have been described elsewhere [23]. KiGGS Wave 1, the first follow-up study, was conducted as a telephone interview among participants of KiGGS baseline after about 6 years (2009–2012), also with newly invited participants aged 0–6 years [24]. Data of the second follow-up study, KiGGS Wave 2, are collected from September 2014 until August 2017 among participants of the baseline survey. In addition, a new sample of 0- to 17-year-olds is drawn, which allows cross-sectional analysis in a representative sample of this age range.

**Fig. 2:**
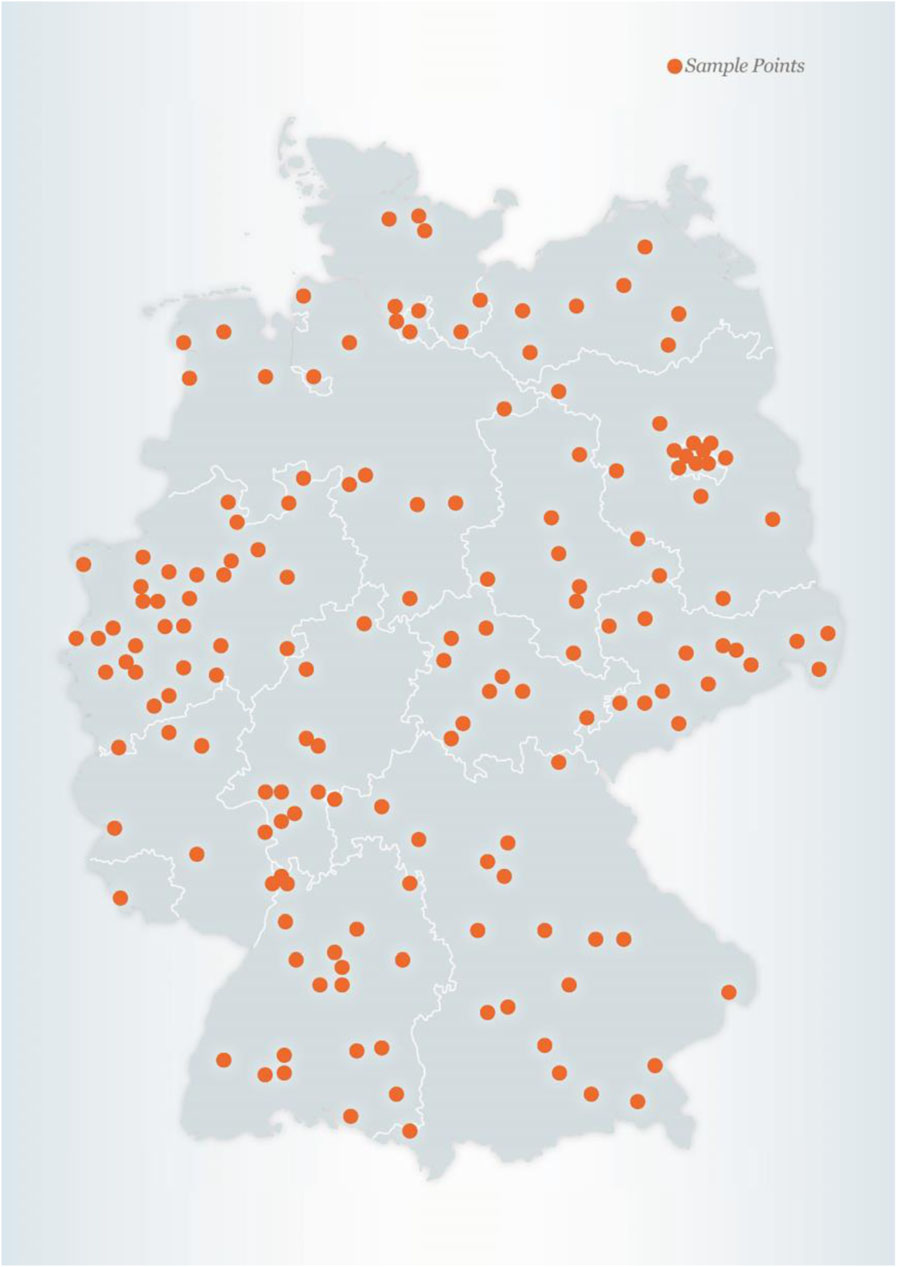
Sample points of KiGGS and EsKiMo II (*N* = 167)

EsKiMo II is conducted within the cross sectional study population of KiGGS Wave 2. Data from about 2400 children and adolescents aged 6–17 will be collected between June 2015 and September 2017 by trained nutritionists of the RKI. Participants of EsKiMo II are a subsample of the KiGGS Wave 2 cross sectional study population who gave their consent to be contacted by further studies. All cross sectional participants who took part in the physical examination of KiGGS Wave 2, plus a random sample of cross sectional participants who only answered the KiGGS questionnaire, are invited to participate in EsKiMo II. All 167 KiGGS sample points (Fig. 2) are visited by trained nutritionists within 3 to 6 months after KiGGS Wave 2. The order of the sample point visits is precisely planned considering all German regions throughout the year to achieve a nationwide representative sample, as well as to avoid seasonal effects.

### Study procedures

About four weeks before a sample point is visited, the selected children and adolescents and their parents receive an invitation letter, including a flyer with comprehensive study information, to participate in EsKiMo II. After a few days, potential participants are contacted by phone by trained interviewers, to provide more information about the background, relevance and study procedures of EsKiMo II. Upon written or oral acceptance, appointments are made within the time frame in which the sample point is visited.

A written informed consent is obtained by parents and all participants over the age of 14 years before data collection. The study is approved by Federal Commissioner for Data Protection and Freedom of Information and by the ethics committee of the Hannover Medical School (Number 2275–2015).

**Fig. 3:**
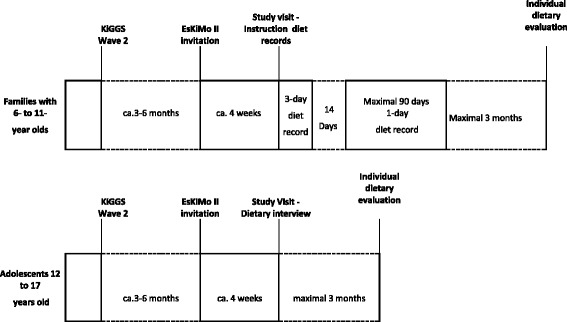
Time schedule of the practical study procedures of EsKiMo II.

Home visits are performed by trained nutritionists. During these visits families with children aged 6–11 are instructed on the completion of weighted food records and adolescents aged 12–7 years are interviewed about their usual dietary intake. A short additional computer assisted interview about dietary habits is conducted for both age groups. All participants receive a monetary incentive upon their participation. In addition, they receive a personal evaluation of the reported food intake, along with corresponding nutrition information, within three months after data collection. Figure 3 gives an overview of the time schedule of the practical study procedures of EsKiMo II.

### Dietary assessment

#### Weighted food records

Parents or legal guardians of children aged 6–11 years receive a standardized instruction by trained nutritionists at home about the completion of the weighted food. If parents or legal guardians are willing to take part in EsKiMo II but have logistical problems (such as time constraints), it is offered to conduct instructions by telephone while the required study materials are provided by post.

Parents or legal guardians of 6- to 11-years-old children have to complete two food records for their child: the first food record over three days has to be completed on predefined random selected days in the week after the home visit and a second food record over one-day should be completed on a randomly selected day between 14 and 90 days later.

For each food consumed, detailed information has to be recorded, by means of exact description of the food, including brand and product names; when applicable, fat content, particularly for milk, cheese and butter; condition at retail (e.g. frozen, conserved, fresh); preparation; kind of packaging (e.g. metal, plastic, paper); and if it is organic. Moreover, the exact time of consumption, the place of preparation, and both the weighted amount served and weighted plate wastes have to be listed. Parents or legal guardians are also asked to report the exact recipes of homemade meals. In addition, deviations from usual food intake caused by illness, celebration days or other special occasions, as well as medication and dietary supplement use, should be reported for each day. For foods consumed out of home, i.e. at schools, restaurants or while staying at friends, a separate food record is provided. It is a short, less detailed version of the weighing record, and when weighting of the amount of food consumed is not possible, portion sizes can be estimated either using a picture book of portion sizes provided or by household measures.

Supporting materials, such as a digital kitchen scale and a picture book [25, 26] for estimation of portion sizes, are provided. The 3-day-weighted food record is given to the families by the interviewer or sent per mail, if the instructions are made by telephone. The separate weighted food record is either provided directly by the interviewer (if it should be completed within the next 21 days after the instruction) or sent by mail.

#### Dietary history interview

Adolescents aged 12–17 years are interviewed face-to-face about their food intake within the past four weeks using the Dietary Interview Software for Health Examination Studies (DISHES). The software was developed at the RKI based on a modified dietary history method to collect data about usual dietary intakes [27]. Portion sizes can be estimated using tableware models as well as a picture book of portion sizes [25, 26]. The DISHES software was updated for EsKiMo II, integrating an actual version of the food portion database and the German Food Code and Nutrient Database Version 3.02 (BLS 3.02).

### Additional questionnaire

In addition to both dietary assessment tools, a computer assisted interview is conducted with the parents or legal guardians of the 6 to 11 year olds or the adolescents themselves to assess additional information, such as in-home and in-school-meals, specific diets, and frequency of consumption of particular food groups. This also includes dietary supplement use within the last four weeks for adolescents, covering the same reference period as DISHES, or within the last 12 months for the 6–11-year-olds. If possible, pictures of the supplement packages are taken to identify the used products.

### Body weight and height

Body weight and height are self-reported and inquired during the DISHES interview for adolescents and during the additional questionnaire for the 6 to 11 year olds.

### Data handling

The completed food records are sent back to the RKI. Upon arrival, they are checked for completeness and plausibility, and, if necessary, inquiries are cleared by telephone. The food records are coded according to a standardized data entry manual by a trained nutritionist using the coding software EAT version 5.3, developed by the Paderborn University. After data entry, data are further handled and checked for plausibility. The amount of each food consumed is calculated as the amount served minus the amount remaining. The composition of self-prepared dishes is calculated from raw ingredients from the reported recipes.

Data from the DISHES interviews are digitally available immediately after the field phase. After some minor corrections based on notes of the interviewers during the field work, plausibility checks are applied and, when applicable, corrections are performed to the data from the DISHES interview. Plausibility checks include the identification of unusual amounts of foods consumed, i.e. extremely low or extremely high amounts, unusual table ware for foods (i.e. meat from a glass) or beverage powder without liquid.

With the consent of participants, a digital audio recording of the DISHES interview is conducted for quality assurance. These recordings are used to improve the overall data quality, since it allows verifying specific data which seem to be implausible. It is also beneficial for training ﻿purposes, interviews quality control, and the systematic recapturation of interviews, assuring standardization over time.

All reported foods are coded using the codes of the German national food composition database BLS 3.02 [28]. Throughout the field phase, information on new foods and products available on the market, which are not yet available in the BLS, is gathered in an additional food database.

For the tableware used in the DISHES interview and for the picture book, a specific food portion size database was developed with corresponding weight amounts for each reported food. The database was used in previous surveys (e. g. GeNuS and EsKiMo I [22]) and is continuously updated during the field phase of EsKiMo II.

The nutrient composition of reported supplements is collected with a supplement database made available by the Max Rubner Institute (MRI). This database was updated for EsKiMo II. The nutrient composition from all dietary supplements reported in EsKiMo II is checked with the available product information from the photographed packages, internet, other available databases, and, if available, by manufacturer information.

To further improve the representativeness of the EsKiMo II data and to correct for deviations in the population structure (e.g. age, sex, community size) between the net study sample and the German population aged 6–17 years in 2015, results will be weighted by specific weighting factors. For the food records, the weighting factor will also be corrected for unbalanced days of the week.

## Discussion

EsKiMo II will provide actual and representative data on the nutrient and food intake of children and adolescents aged 6–17 years living in Germany. It enables to identify changes in children and adolescents’ dietary behavior in the last 10 years by comparison with the EsKiMo I data, and risk groups either with insufficient nutrient intake or unhealthy eating habits.

A major strength of EskiMo II is that the study is performed within the framework of KiGGS Wave 2, allowing a combination of data from both studies. This enables many analyses, i.e., the association of dietary intake with sociodemographic data from KiGGS. In addition, most participants of EsKiMo II participated on the physical examination of KiGGS Wave 2. For these participants with health and laboratory data available, a comprehensive analysis between nutrition and health characteristics is possible. However, the time lag between KiGGS wave 2 and EsKiMo II of 3 to 6 months should be considered. Consequently, the analyses of EsKiMo II data in relation to KiGGS Wave 2 should focus on variables reflecting long term behavior or conditions.

A further advantage of EsKiMo II is the use of established dietary assessment instruments to collect data. The dietary record method is recommended for dietary studies with children up to the age of 10 years [21]. However, this method assesses the current short term diet rather than the usual diet. In EsKiMo II, a separated randomly selected day is assessed in addition to the three day weighted food record, allowing a better estimation of the usual dietary intake. The same methodology is also applied in a further nutrition module of KiGGS Wave 2, the German food survey for infants, toddlers, and children (KiESEL study – 0-6 year olds) [29], conducted in about the same time period by the BfR. Hence, the﻿ dietary intake data from 6 to 11 year old participants from EsKiMo II and KiESEL participants will be highly comparable. The dietary history software DISHES was developed to be used within surveys and the interview can be carried out relatively quickly in comparison to traditional diet history methods, requiring a one-time contact with the participants. During the interview, data is directly coded into a database. Therefore, the data analysis can start relatively quickly once the data collection and preparation has been completed. Besides, DISHES was also used in EsKiMo I and has also been already validated for adults [27] and used in several surveys, e.g. the NVS II [30].

Another important strength of the study is that dietary assessment methods for EsKiMo II are very similar to EsKiMo I, which guarantees a good comparability between both studies. However, there are some limitations, which have to be considered when comparing data from EsKiMo II with EsKiMo I. The first limitation considers the use of different versions of the German national food composition database in both nutrition surveys. Food coding is based on BLS 2.3 in EsKiMo I and on BLS 3.02 in EsKiMo II. As intake of energy and most nutrients may differ according to the BLS version used to calculate the intakes [31], a re-coding of the data from EsKiMo I from BLS 2.3 to BLS 3.02 is necessary before analyzing any changes between EsKiMo I and EsKiMo II. This will improve the comparability since several necessary corrections have been made ﻿b﻿etween both versions, although some changes may also reflect real changes in composition of foods within this decade. The second limitation considers the assessment of dietary intake of 6- to 11-year-old children, which is based on weighted food records in EsKiMo II, instead of on an estimated food record in EsKiMo I. Weighted food records may improve the precision of consumed portion sizes, which is especially important for risk evaluations of the Federal Institute for Risk Assessment (BfR). However, it will be difficult to exclude bias from estimated portion sizes from EsKiMo I and weighted portion sizes in EsKiMo II, when comparing both studies. Sensitivity analysis in subsamples with both measured and estimated portion sizes may enable to discriminate between real differences and differences due to the kind of estimation of portion sizes. The third limitation considers the extension of the dietary assessment by an independent one-day food record in EsKiMo II in addition to the three day food records applied in both studies.

Data from EsKiMo II will be analyzed from 2018 onwards. Overall, data from EsKiMo II will update the current knowledge of the food and nutrient intake of children and adolescents in Germany. Results of EsKiMo II are relevant for decisions, measures, and evaluations within nutrition, consumer, and health policy in Germany, as it may identify deficits in food and nutrient intake, as well as its determinants and risk groups.

## Abbreviations

BfR: Federal Institute for Risk Assessment
BLS: German Food Code and Nutrient Database
BMEL: German Federal Ministry of Food and Agriculture
DISHES: Dietary Interview Software for Health Examination Studies
EFSA: European Food Safety Agency
EsKiMo: Eating Study as a KiGGS module
KiESEL: German food survey for infants, toddlers and children
KiGGS: German Health Interview and Examination Survey for Children and Adolescents
MRI: Max Rubner Institut
NVS II: German National Nutrition Survey II
RKI: Robert Koch Institute
